# Preparation of Starch-Hard Carbon Spherules from Ginkgo Seeds and Their Phenol-Adsorption Characteristics

**DOI:** 10.3390/molecules23010096

**Published:** 2018-01-02

**Authors:** Hongxia Chen, Chengzhang Wang, Jianzhong Ye, Hao Zhou, Ran Tao, Wenjun Li

**Affiliations:** 1 Institute of Chemical Industry of Forest Products, Chinese Academy of Forestry, Nanjing 210042, China; shirenyahui@126.com (H.C.); Yejianzhong@163.com (J.Y.); zhouhaolhs@163.com (H.Z.); trmoon1949@126.com (R.T.); lwj88611@163.com (W.L.); 2 Key and Open Laboratory on Forest Chemical Engineering, State Forestry Administration of the People’s Republic of China, Nanjing 210042, China; 3 Institute of New Technology of Forestry, Chinese Academy of Forestry, Beijing 10091, China

**Keywords:** ginkgo seeds, starch, spherule, adsorption, phenols

## Abstract

Carbon spherules from ginkgo seed starch were prepared through stabilization and carbonization processes. The ginkgo seed starch was first stabilized at 195 °C for 18 h, then carbonized at 500 °C for 2 h under an N_2_ atmosphere. The characterization results confirmed that carbon spherules were in the size range of 10–20 μm. Experimental data were also evaluated to find out the kinetic characteristics of phenols on the carbon spherules during the adsorption process. Adsorption processes for phenol, *p*-nitrophenol and *p*-chlorophenol were found to follow the pseudo-first order kinetic model with *R*^2^ values of 0.995, 0.997 and 0.998, while the rate constants *k*_1_ = 0.014, 0.009 and 0.011 min^−1^ showed that the adsorption is mainly controlled by adsorbate diffusion. The equilibrium data were analyzed with the Langmuir, Freundlich and Temkin–Pyzhev models and the best fit was observed with the Freundlich isotherm, suggesting the physical adsorption of phenols. From the thermodynamic functions, ∆G, ∆H, and ∆S were calculated, which showed that adsorption is more favorable at low temperature and is an exothermic process, and the adsorption of *p*-nitrophenol and *p*-chlorophenol were more advantageous than that of phenol.

## 1. Introduction

Ginkgo seeds for edible use and medication have 3000 years of history in China. Ginkgo seed is rich in protein, amino acid, fat, sugar, vitamin C, riboflavin, flavonoids and lactones etc., and have been used for lowering blood cholesterol, improving the blood rheology and treating pulmonary diseases [1]. At present, ginkgo seeds are eaten after stir-frying, baking and cooking, and in addition way are used in the development of candied fruit, canned foods and functional drinks. However, eating ginkgo seeds can result in allergic reactions in humans [2], which limits their high-value application in the food industry.

The ginkgo seed contains 60–70% starch, 10–20% protein, 2–4% lipid and 0.8–1.2% pectin. The starch from the ginkgo seed consists of 20–30% linear amylose and 70–80% highly-branched amylopectin [3,4]. Native starch has limited functionalities and is commonly modified to expand the functional range. Waxy maize starch nanocrystals obtained by hydrolysis of native granules have been used as a reinforcing agent in a thermoplastic waxy maize starch matrix plasticized with glycerol [5]. Pea starch nanocrystals have been incorporated into a soy protein isolate matrix to produce a class of fully biodegradable nanocomposites [6].

Starch is a good biomass candidate for the preparation of some kinds of carbon material [7,8]. Carbon materials are a preferred adsorbent for the removal of micropollutants from the aqueous phase [9,10,11]. Phenol and phenolic compounds are present in wastewaters from coal-related industries. Owing to their inherent toxicity and good solubility, phenols are considered to be one of the potential organic pollutants being discharged into the environment, causing severe physiological disorders.

In the present work, starch-hard carbon spherules from ginkgo seeds were prepared by stabilization and carbonization processes under a nitrogen atmosphere. Methods such as infrared spectroscopy (IR), thermogravimetry (TG), scanning-electron microscopy (SEM) and X-ray diffraction (XRD) were applied to characterize the starch and carbon spherules of the ginkgo seeds. Then the starch-hard carbon spherules were used as an absorbent for the removal of phenols from aqueous solution. The kinetic, thermodynamic, and equilibrium isotherm data were evaluated with several models to understand the adsorption mechanisms. Based on the experimental results, a mechanism for phenol removal from aqueous solution was proposed. The main advantage of using starch-hard carbon spherules is the ease of preparation, low cost and large adsorption capacity.

## 2. Results

### 2.1. Specific Surface Area and Pore Structure

**Figure 1:**
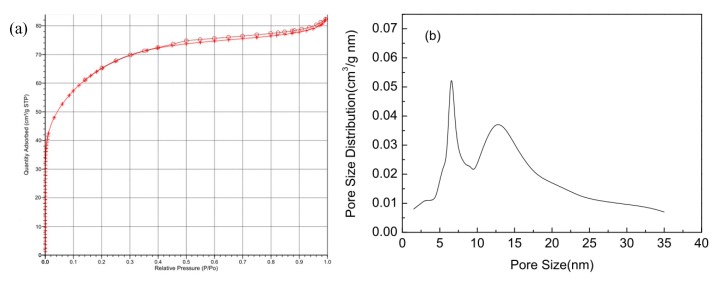
(**a**) N_2_ adsorption-desorption isotherms; and (**b**) pore-size distributions for ginkgo seed starches.

Figure 1a,b shows the N_2_ adsorption–desorption isotherms and pore size distributions for ginkgo seed starches. The samples exhibit a combination of type I and IV, indicating the coexistence of micropores and mesopores. The specific surface area of ginkgo seed starches was found to be 245 m^2^/g. The pore-size distribution curve for the ginkgo seed starches is mostly centered in the region of 5–15 nm.

### 2.2. Elemental Analysis

The elemental analysis of native, stabilized and carbonized ginkgo seed starches are shown in Table 1. The carbon, hydrogen and oxygen ratios were 44.79%, 6.18% and 48.76% in the native ginkgo starches. However, after stabilization and carbonization, carbon concentration increased due to the occurrence of decomposition and condensation reactions in the native ginkgo starch.

### 2.3. Fourier-Transform Infrared (FT–IR) Spectroscopy

**Figure 2:**
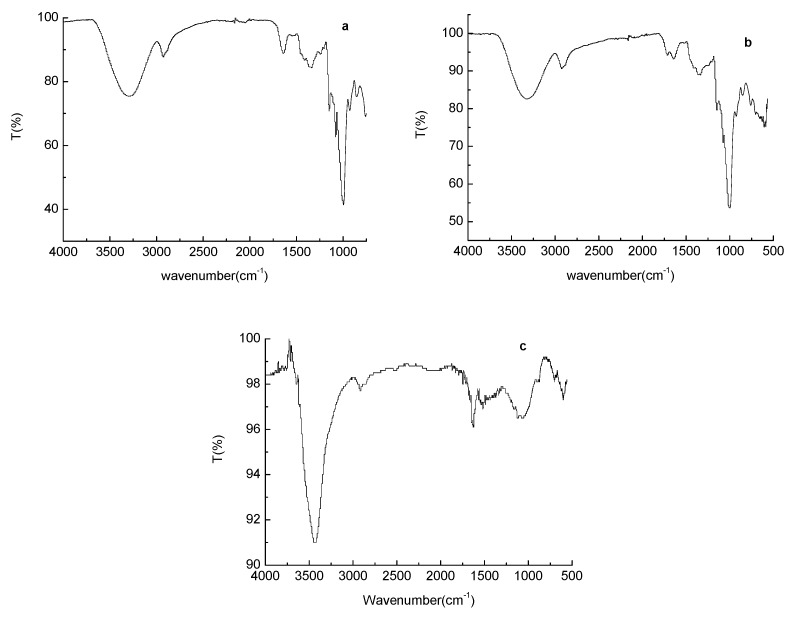
Fourier-transform infrared (FT–IR) spectroscopies of native (**a**); stabilized (**b**); and carbonized (**c**) ginkgo seed starches.

Figure 2 shows the Fourier-transform infrared (FT–IR) spectra of native, stabilized and carbonized ginkgo seed starches. Both native and stabilized ginkgo seed starches showed similar FT–IR spectra results, with the bands at 3412 cm^−1^ and 1158 cm^−1^ corresponding to the O–H (hydroxyl or carboxyl) stretching vibration and C–OH bending vibration, indicating that large numbers of –OH groups are on the native and stabilized ginkgo seed starch surface. The peaks at around 2924 cm^−1^ and 1645 cm^−1^ were associated with –CH_2_ stretching vibrations and deformation vibrations of the hydroxyl groups in water, respectively. However, after carbonization, the absorption peaks of C–H, C–O and –OH become weaker, which indicated that the functional groups of ginkgo seed starch were pyrolyzed by the high temperature.

### 2.4. Scanning-Electron Micrograph (SEM) Observation

**Figure 3:**
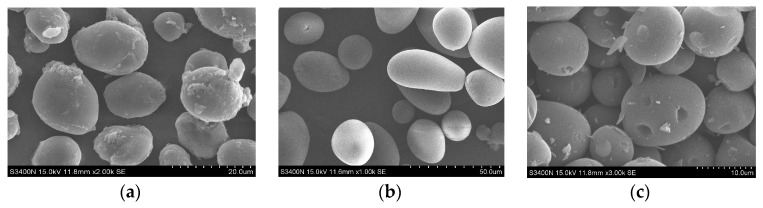
Scanning-electron micrograph (SEM) images of native (**a**); stabilized (**b**); and carbonized (**c**) ginkgo seed starches.

The scanning-electron micrographs (SEM) of starch and starch-hard carbon spherules of ginkgo seeds are shown in Figure 3. The ginkgo seed starch granules were oval or spherical in shape with sizes ranging from 10–20 μm, with smooth surfaces, and some granules had dents on the surface. Starch-hard carbon spherules were spherical in shape with sizes ranging from 5–10 μm, with smooth surfaces. The size of the carbon spherule was less than that of the starch, this is because that the internal structure of the starch particles shrank after the heat reaction, a phenomenon that has been reported by Shu [8].

### 2.5. X-ray Diffraction (XRD) Characterization

**Figure 4:**
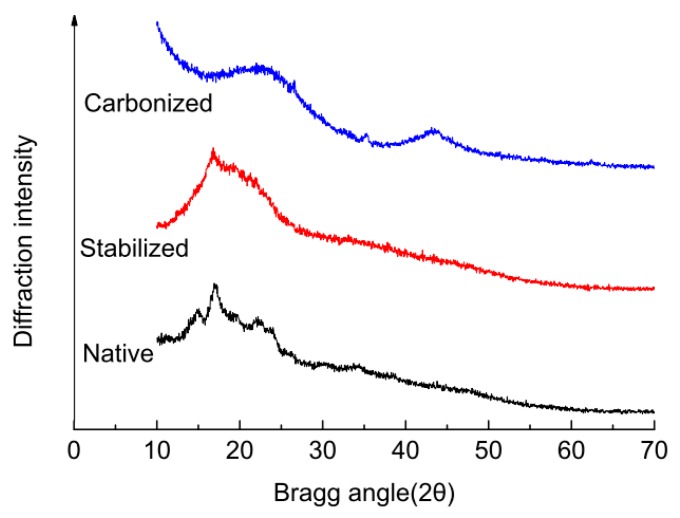
X-Ray Diffraction (XRD) figurations of native, stabilized and carbonized ginkgo seed starches.

X-Ray Diffraction (XRD) was used to obtain structural information about native, stabilized and carbonized ginkgo seed starches, and the results are shown in Figure 4. In the XRD pattern of native ginkgo seed starch, there are three characteristic peaks at 2θ = 15.16°, 16.92° and 21.68°. This is direct evidence that ginkgo seed starch belongs to C-type crystallite starch [2]. However, the three characteristic peaks disappear completely after the potato starch has been stabilized at 195 °C for 18 h and, instead, a broad diffraction peak appears at about 2θ = 16.80°. This indicates that the regular supramolecular structure of the ginkgo seed starch has been destroyed, which may be caused by the loss of water [4]. After carbonization, a significant change happened to the X-ray diffraction patterns of carbonized ginkgo seed starch: two weak and broad diffraction peaks(2θ = 23.64°, 44.13°) appear, a main peak located at about 23.64° corresponding to the diffraction caused by the 002 planes, and a lower intensity peak at 44.13° due to 100 planes [12]. The disappearance of the characteristic peaks of ginkgo seeds indicated that the regular molecular structure of the ginkgo seed starch had been destroyed. This may be caused by the loss of the water contained in ginkgo seed starch, according to the results of FT–IR and elemental analysis. High-diffraction intensity existed in the small angle area, but forms a trailing style in the low angle area, which reflected the micropore structure inside the material [13].

### 2.6. Adsorption Kinetics of Phenols on the Carbon Spherules

**Figure 5:**
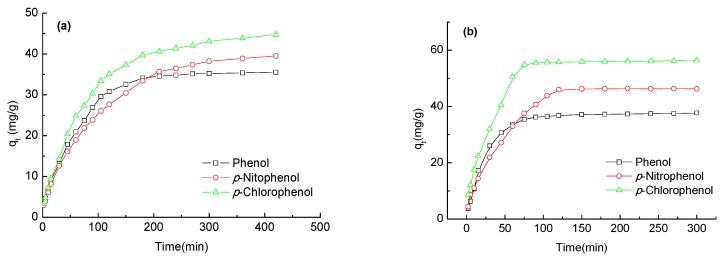
Kinetic adsorption curves of phenol compounds onto ginkgo seed starch-hard carbon spherules (**a**); and coal-activated carbon (**b**).

The evaluation of kinetics is necessary to assess adsorption performances of phenolic compounds (phenol, *p*-nitrophenol and *p*-chlorophenol). Adsorption kinetics of phenols on the ginkgo seed starch-hard carbon spherules were studied at 298.15 K. The effect of contact time on phenols with carbon spherules and coal-activated carbonare presented in Figure 5. Figure 5 shows that the adsorption capacity of phenols on carbon spherules and coal-activated carbon increased with the extension of adsorption time until equilibrium. The adsorption capacity on carbon spherules rapidly increased in the first 120 min, then slowly increased, finally reaching equilibrium after 210 min. The adsorption capacities of phenol, *p*-nitrophenol and *p*-chlorophenol on carbon spherules were 35.55 mg/g, 39.5 mg/g and 44.75 mg/g, respectively. The adsorption capacities of phenol, *p*-nitrophenol and *p*-chlorophenol on coal-activated carbon were 37.68 mg/g, 46.3 mg/g and 56.38 mg/g, respectively. The adsorption capacity of phenols on coal-activated carbon were higher than carbon spherules, and this may be due to the specific surface area and pore size. However, the equilibrium times of *p*-nitrophenol and *p*-chlorophenol on carbon spherules were longer than that of phenol, and the adsorption capacity for *p*-nitrophenol on carbon spherules rapidly increased in the first 210 min. Thereafter, it proceeded at a slower rate from 210 min to 420 min. The adsorption capacity for *p*-chlorophenol on carbon spherules rapidly increased in the first 180 min, then increased slowly from 180 min to 420 min. Therefore, the adsorption of phenol, *p*-nitrophenol and *p*-chlorophenol on carbon spherules belongs to the slow-adsorption type [14].

**Figure 6:**
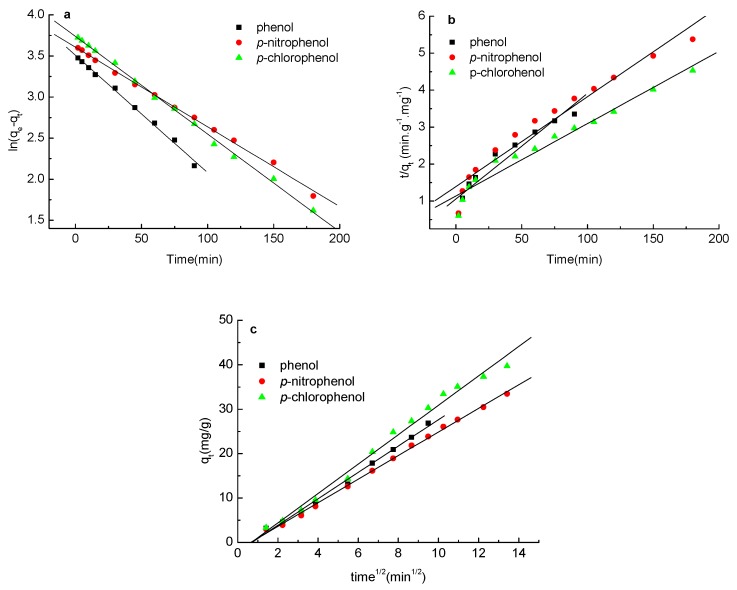
(**a**) Pseudo-first order adsorption kinetics plot; (**b**) pseudo-second order adsorption kinetics plot; and (**c**) Weber–Morris adsorption kinetics plot.

In order to gain a better understanding of the adsorption process, a pseudo-first-order kinetic model (Equation (6)), pseudo-second order kinetic model (Equation (7)) and Weber–Morris model (Equation (8)) have been used to fit the experimental data. The best-fit model was selected on the basis of the linear regression correlation coefficient values (*R*^2^). Compared with experimental results, the kinetic data were adequately fitted with the pseudo-first order kinetic model and exhibited good linearity with the *R*^2^ values of 0.995, 0.997 and 0.998; and the value of *q*_e_ was in accordance with the value gained experimentally (Figure 6a). The rate constant (*k*_1_) and theoretical *q*_e_ values are given in Table 2, and it is clear from these results that the experimental and theoretical *q*_e_ values are in accordance with each other. The Weber–Morris model was also used to describe the adsorption process of phenol and *p*-nitrophenol (*R*^2^ > 0.99) (Figure 6c). Whereas the pseudo-second order model fitted the kinetic data poorly, having *R*^2^ values of 0.926, 0.952 and 0.960, there was a great difference between the adsorption capacity calculated from the model and that obtained experimentally (Figure 6b). The correlation coefficient value of the pseudo-second order kinetic equation is little lower than the pseudo-first order kinetic equation, indicating that this adsorption is mainly controlled by adsorbate diffusion, rather than surface control. Furthermore, the principle of pseudo-first order kinetics assumed that the rate-limiting step might be the adsorption, which is in agreement with physical adsorption being the rate-controlling step. This indicated that the adsorption of phenols onto the carbon spherules was mainly physical adsorption. From the apparent rate constant *k*_1_, the adsorption rate of phenol onto the carbon spherules was faster than *p*-nitrophenol, which indicated that the molecular volume of the phenols was the main influencing factor in the adsorption process. The molecular volumes of *p*-nitrophenol and *p*-chlorophenol were much larger than that of phenol. Compared with phenol, high intraparticle transfer resistance existed in the process of *p*-nitrophenol diffusion into the carbon spherules.

### 2.7. Adsorption Isotherms of Phenols on the Carbon Spherules

**Figure 7:**
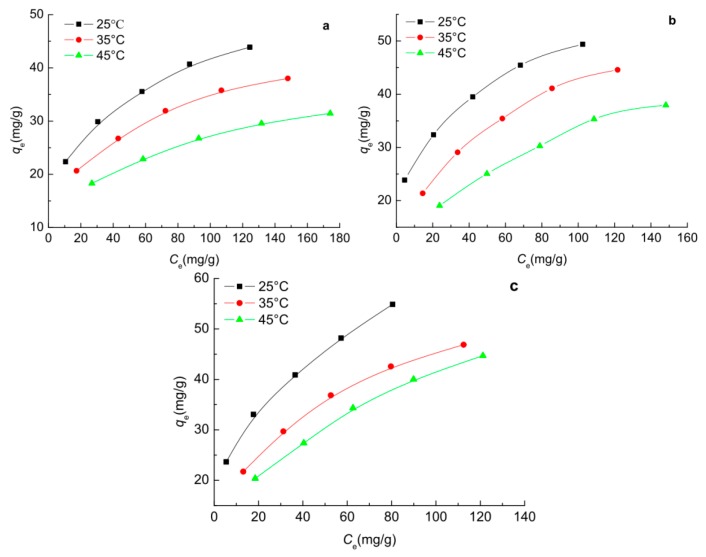
Equilibrium adsorption isotherms of phenol (**a**); *p*-nitrophenol (**b**); and *p*-chlorophenol (**c**) onto ginkgo seed starch-hard carbon spherules.

The equilibrium adsorption isotherms of phenol, *p*-nitrophenol and *p*-chlorophenol from aqueous solution onto the ginkgo seed starch-hard carbon spherules were obtained at 25 °C, 35 °C, and 45 °C, respectively, as shown in Figure 7. It was obvious that the adsorption capacities for phenols onto carbon spherules increased with an increase in the equilibrium concentration of phenols at the given temperature, as in Figure 7a–c. Furthermore, the adsorption capacities for phenols onto carbon spherules decreased with an increase in the experimental temperature, which might suggest an exothermic process.

In order to gain a better understanding of the adsorption mechanism of the phenols onto carbon spherules, a Langmuir model (Equation (9)), Freundlich model (Equation (10)) and Temkin–Pyzhev model (Equation (11)) have been used to fit the experimental data. From a comparison of the *R*^2^ values given in Table 3, it can be concluded that in all cases for the adsorption of phenols to the carbon spherules, the Freundlich equation represents a better fit to the experimental data than the Langmuir and Temkin–Pyzhev equations. The Freundlich isotherm equation reasonably demonstrates the adsorption process of phenol, *p*-nitrophenol and *p*-chlorophenol. The 1/*n* was smaller than 1 (values of *n* were 3.623, 3.425, 3.390, 4.219, 2.841, 2.577, 3.257, 2.747 and 2.347, respectively), which indirectly suggests a favourable physical adsorption [15], indicating a distribution of surface sites or any factor that causes a decrease in adsorbent–adsorbate interaction with increasing surface density. Considering the existence of chemical adsorption, the Langmuir isotherm model was demonstrated on the monolayer adsorption, Table 3 and this showed that it also fitted well for phenol and *p*-nitrophenol. The *q*_m_ obtained from the slope of the linear plot were 50, 55.56, 62.50 mg/g for phenol, *p*-nitrophenol and *p*-chlorophenol, respectively. The enhanced adsorption performance of the *p*-chlorophenol was directly relative to the distribution coefficient between capryl alcohol and water [16]. The hydrophobic property of *p*-chlorophenol also led to higher adsorption performance. It was also shown that the adsorption isotherm data fit the Temkin–Pyzhev isotherm model poorly, with a lower *R*^2^ value compared to the Freundlich and Langmuir isotherm models (Table 3).

### 2.8. Adsorption Thermodynamics of Phenols on the Carbon Spherules

The decreasing adsorption capacities for phenols onto carbon spherules with an increase in the experimental temperature can be attributed to the exothermic process. However, this needed to be further explained through evaluation of the thermodynamic parameters. The ∆*G*, ∆*H*, and ∆*S* associated with the adsorption process were estimated according to the following equations:

(1) $$K=M/K_{L}$$

(2) ΔG=−RTlnK

(3) lnK=−ΔH/RT+ΔS/R

where *M* is the molecular weight of phenol, *p*-nitrophenol and *p*-chlorophenol (g/mol); *R* is the gas constant (8.314 J/(mol K)); and *T* is the absolute temperature (*K*).

The thermodynamic parameters are listed in Table 4. The ∆*G* values of the adsorption processed of phenols onto the carbon spherules were negative at the investigated temperatures (25 °C, 35 °C, 45 °C), which indicated that the adsorption processes were spontaneous. The ∆*G* values of *p*-nitrophenol and *p*-chlorophenol were larger than those of phenol on corresponding carbon spherules, meaning that the adsorption of *p*-nitrophenol and *p*-chlorophenol were more advantageous than that of phenol [17]. The ∆*H* for the adsorption of phenols were negative, indicating that the processes were exothermic, and the adsorption is favored and gets easier at low temperature. The values of ∆*S* were found to be negative due to the phenols on the carbon spherules, making it well ordered, and also show the affinity of carbon spherules for phenols in aqueous solution [18]. Hence, 25 °C was found to be the favorable temperature for the adsorption of phenols onto carbon spherules.

## 3. Materials and Methods

### 3.1. Materials

The ginkgo seeds of *Ginkgo biloba* L. (longyan) purchased from Taixing, China, were harvested in November 2015. Phenol, *p*-nitrophenol and *p*-chlorophenol were purchased from Sigma-Aldrich Co., Ltd., (Shanghai, China). Sodium hydroxide, ethanol, methanol, acetic acid, hydrochloric acid and ammonium potassium dihydrogen phosphate were purchased from Sinopharm Chemical Reagent Co., Ltd., (Nanjing, China).

### 3.2. Starch Preparation

The ginkgo seeds were shelled manually, and the nuts were dried at room temperature for two weeks, then crushed into a powder. The starch was extracted from the nuts according to the procedure of Wang [19]. The starch method was under 4 min of sonification at 500 W and a seed powder/water ratio of 1.1 (g/mL) was prepared. The mixed solution was filtered through a 150 μm sieve and then centrifuged at 3000× *g* for 10 min. The sediment was washed thoroughly with distilled water and ethanol, then dried by vacuum drying at 40 °C for 24 h.

### 3.3. Starch-Hard Carbon Spherule Preparation

The ginkgo seed starches were prepared by a two-step process including stabilization and carbonization. The stabilization temperature was maintained at 195 °C for 18 h. Then, the carbonization of the stabilized samples was carried out under a nitrogen atmosphere at 500 °C for 2 h, with a heating rate of 2 °C/min.

### 3.4. Structural Characterization

The morphologies of the ginkgo seed starch-hard carbon spherules were observed by a scanning electron microscope (SEM, S-3400N, Hitachi Limited, Tokyo, Japan). The structure characterizations were performed by Fourier-transform infrared spectroscopy (FT–IR, Nicolet iS10, Thermo electron corporation, Waltham, MA, USA). X-ray diffraction was performed on a RigakuD/Max2500 X-ray diffractometer using CUKα radiation (40 kV, 200 mA, λ = 1.54056 nm).

### 3.5. Static Adsorption Experiments

Each carbon spherule was accurately weighed (0.1 g) and carefully transferred into a 100 mL flask. After 25 mL phenol solution was added, the flask was put into a water bath shaker (120 rpm) and shaken at 25 °C for 3 h. The final concentrations of phenols in the solution were measured by UV (280 nm). Static desorption was carried out as follows: the supernatant was removed by filtering and the carbon spherule was added into a 100 mL flask which was shaken at 150 rpm, 25 °C for 3 h. The adsorption of phenols onto carbon spherules was quantified by the following equations:

(4) $$q_{e}=\frac{(C_{0}-C_{e})\times V_{i}}{W}$$

(5) $$F=\frac{C_{0}-C_{e}}{C_{0}}\times 100\%$$

where *q*_e_ is the adsorption capacity, mg/g; *F* is the adsorption ratio (%); *C*_0_ is the initial concentrations of phenols (mg/L); *C*_e_ is the equilibrium concentrations of phenols in the solutions (mg/L); *V*_i_ is the volume of the initial solution (mL); and *W* is the weight of the dry carbon spherule (g).

### 3.6. Adsorption Kinetics

The adsorption kinetic curves of phenols on the carbon spherules were studied according to the following operation mode: 25 mL of phenol solution (*C*_0_ = 200 mg/L) was brought in contact with the carbon spherules (0.1 g) in a 100 mL conical flask, and the flask was put into a water bath shaker(120 rpm) at 25 °C. The concentration of phenols in the adsorption solution was determined by high-performance liquid chromatography (HPLC) at different times until equilibrium.

The pseudo-first order kinetics, pseudo-second order and Weber–Morris models were applied to illustrate the adsorption mechanisms of phenols onto the carbon spherules. The best-fit model was selected on the basis of the linear regression correlation coefficient values (*R*^2^).

The equation of the pseudo-first order kinetics model is:

(6) $$\ln(q_{e}-q_{t})=\ln q_{e}-k_{1}t$$

The equation of the pseudo-second order kinetics model is:

(7) $$\frac{t}{q_{t}}=\frac{1}{k_{2}q_{e}{}^{2}}+\frac{1}{q_{e}}t$$

The equation of the Weber–Morris model is:

(8) $$q_{t}=k_{p}t^{1/2}+C$$

where *q*_e_ and *q*_t_ are the adsorption capacity for phenols onto the carbon spherules at equilibrium and at any time *t* (mg/g carbon spherules), respectively; and *C* is the intercept for the curve of *q*_t_ versus *t*^1/2^. The parameters *k*_1_ (1/min), *k*_2_ (g/(mg min)) and *k*_p_ are the rate constants of the pseudo-first order, pseudo-second order and Weber–Morris models for the adsorption process, respectively.

### 3.7. Adsorption Isotherm

Extract solutions (25 mL) with different concentrations of phenols (*C*_0_ = 100, 150, 200, 250 and 300 mg/L) were brought into contact with carbon spherules (0.1 g) in 100mLconical flasks. The flasks were continually shaken for 5 h at temperatures of 25 °C, 35 °C and 45 °C. Then, the phenol concentrations of the adsorption solutions were analyzed by HPLC.

The Langmuir isotherm, Freundlich isotherm and Temkin–Pyzhev isotherm models were chosen to optimize the adsorption isotherm by comparing the values of correlation coefficients.

The equation of the Langmuir isotherm model is:

(9) $$\frac{C_{e}}{q_{e}}=\frac{K_{L}}{q_{m}}+\frac{C_{e}}{q_{m}}$$

where *q*_e_ and *q*_m_ are the equilibrium and maximum adsorption capacity (mg/g carbon spherules), respectively; *C*_e_ is the equilibrium concentration of the phenol solution (mg/L); and *K*_L_ is the parameter related to the adsorption energy (L/mg).

The equation of the Freundlich isotherm model is:

(10) $$\mathrm{lg}(q_{e})=(\frac{1}{n})\mathrm{lg}C_{e}+\mathrm{lg}K_{F}$$

where *K*_F_ reflects the adsorption capacity of an adsorbent ((mg/g)(L/mg)1/*n*). The parameter *n* represents the adsorption affinity of the adsorbent for an adsorbate. The parameters *q*_e_ and *C*_e_ are the same as mentioned above.

The equation of the Temkin–Pyzhev isotherm isotherm model is:

(11) $$q_{e}=(\frac{RT}{b_{T}})\ln C_{e}+(\frac{RT}{b_{T}})\ln A^{\prime }$$

where *R* is the gas constant (8.314 J/(mol K)); *T* is theabsolute temperature (K); and *A*′ and *b*_T_ are the isotherm constant (L mg^−^^1^) and Temkin–Pyzhev constant (J/mol), respectively. The parameter *q*_e_ is the same as mentioned above.

### 3.8. High-Performance Liquid Chromatography (HPLC) Analysis of Phenols

The extractions were analyzed with HPLC. Chromatographic analyses were carried out on a Shimadzu HPLC system (Kyoto, Japan) with an RP-18 endcapped column (4.6 × 250 mm, 5 μm). The mobile phase was methanol–water (5:5, *v*/*v*). The flow rate was maintained at 1 mL/min. Injection was performed via a 20-μL injector. Sample separation was monitored at UV 270 nm.

## 4. Conclusions

In summary, the ginkgo seed carbon spherules were successfully prepared and characterized. The ginkgo seed starch was first stabilized at 195 °C for 18 h, then carbonized at 500 °C for 2 h under a N_2_ atmosphere, and the size range was 10–20 μm. The results suggest that the adsorption kinetics of phenol, *p*-nitrophenol and *p*-chlorophenol onto carbon spherules follow a pseudo-first order kinetic model (*R*^2^ = 0.995, 0.997 and 0.998), indicating the physical adsorption of phenols onto carbon spherules. From the apparent rate constant *k*_1_, the adsorption rate of phenol onto the carbon spherules was faster than *p*-nitrophenol, which indicated that the molecular volume of the phenols was the main influencing factor in the adsorption process. The phenol adsorption of carbon spherules’ adsorbent fitted with the Freundlich model well (*R*^2^ > 0.99). The thermodynamic functions of ∆*G*, ∆*H*, ∆*S* were calculated in the process of the carbon spherules absorbing phenols. They were ∆*H* = −23.445 kJ/mol, −54.249 kJ/mol and −43.050 kJ/mol of phenol, *p*-nitrophenol and *p*-chlorophenol, ∆*G* < 0, and ∆*S* < 0, respectively, which indicated that the phenol adsorption is an exothermic process, and is more favorable towards lower temperatures. This research may provide ideas for exploiting ginkgo seeds and be conducive to improved water treatment.

## Figures and Tables

**Table 1:** Elemental analyses of native, stabilized and carbonized ginkgo seed starches.

Kind	C (Weight%)	H (Weight%)	O (Weight%)
Ginkgo starch	44.79	6.18	48.76
Stabilized ginkgo starch	72.24	6.12	21.29
Carbonized ginkgo seed starch	93.58	4.34	0.85

**Table 2:** Kinetic adsorption parameters of phenols onto ginkgo seed starch-hard carbon spherules.

Adsorbate	Pseudo-First Order Equation			Pseudo-Second Order Equation			Weber–Morris	
	*k*_1_ (1/min)	*q*_e_ (mg/g)	*R*_1_^2^	*k*_2_ (g/mg·min)	*q*_e_ (mg/g)	*R*_2_^2^	*k*_p_ (mg/min^1/2^/g)	*R*_p_^2^
Phenol	0.014	33.41	0.995	7.42 × 10^−4^	35.71	0.926	2.971	0.994
*p*-Nitrophenol	0.009	36.86	0.997	4.13 × 10^−4^	41.67	0.952	2.656	0.997
*p*-Chlorophenol	0.011	42.01	0.998	3.18 × 10^−4^	52.63	0.960	3.310	0.989

**Table 3:** Evaluation constants for the adsorption of phenols onto ginkgo seed starch-hard carbon spherules from the Langmuir, Freundlich and Temkin–Pyzhev equations.

Adsorbate	T (°C)	Langmuir			Freundlich			Temkin-Pyzhev		
		*R*_L_^2^	*q*_m_ (mg/g)	*K*_L_ (L/mg)	*R*_F_^2^	*n*	*K*_F_ (mg/g)	*R*_T_^2^	*b*_T_ (L/mg)	*A*′
Phenol	25	0.993	50.00	17.6	0.998	3.623	11.64	0.988	282.26	1.107
	35	0.996	45.45	24.68	0.996	3.425	8.97	0.992	308.19	0.651
	45	0.996	37.34	31.89	0.997	3.390	6.92	0.994	369.48	0.457
*p*-Nitrophenol	25	0.990	55.56	11.00	0.994	4.219	16.368	0.971	301.45	3.357
	35	0.994	55.56	26.83	0.997	2.841	8.375	0.992	230.60	0.442
	45	0.991	50.00	43.40	0.995	2.577	5.546	0.986	248.83	0.234
*p*-Chlorophenol	25	0.979	62.50	13.81	0.994	3.257	13.836	0.962	221.13	1.290
	35	0.993	58.82	26.29	0.997	2.747	8.531	0.991	215.83	0.440
	45	0.987	58.82	41.06	0.997	2.347	5.808	0.981	202.07	0.232

**Table 4:** Thermodynamic parameters for adsorption process of phenols on the carbon spherules at different temperatures.

Thermodynamic Parameters	∆*G* (kJ/mol)			∆*H* (kJ/mol)	∆*S* (J/(mol·K))
	25 °C	35 °C	45 °C		
Phenol	−4.234	−3.510	−2.945	−23.445	−64.519
*p*-Nitrophenol	−6.289	−4.216	−3.081	−54.249	−161.366
*p*-Chlorophenol	−5.530	−4.066	−3.019	−43.050	−126.038

## References

[1] Mahady G.B. (2001). Global harmonization of herbal health claims. J. Nutr..

[2] Yang J.T., Wu C.E. (2009). Advance in allergic composition and mechanism of allergy of *Ginkgo biloba* seeds. Food Sci. Technol..

[3] Miao M., Jiang H., Jiang B. (2012). Structure and functional properties of starches from Chinese ginkgo (*Ginkgo biloba* L.) nuts. Food Res. Int..

[4] Yilmaz M.T., Karaman S., Kayacier A. (2013). Mathematical approach for two component modeling of salep-starch mixtures using central composite rotatable design: Part I. Physicochemical and steady shear properties. Food Hydrocoll..

[5] Angellier H., Molinaboisseau S., Dole P. (2006). Thermoplastic starch-waxy maize starch nanocrystals nanocomposites. Biomacromolecules.

[6] Zheng H., Ai F., Chang P.R. (2010). Structure and Properties of Starch Nanocrystal-Reinforced Soy Protein Plastics. Polym. Compos..

[7] Zhou S., Wang C.Y., Chen M.M., Shi Z.Q., Liu N. (2010). Preparation of carbon spheres from potato starch and its stabiliztion mechanism. New Carbon Mater..

[8] Shu J., Shui M., Xu D. (2011). Preparation of nano-sized hard carbon spherule and its electrochemical property. J. Electroanal. Chem..

[9] Khezami L., Capart R. (2005). Removal of chromium(VI) from aqueoussolution by activated carbons: Kinetic and equilibrium studies. J. Hazard. Mater..

[10] Lorenc-Grabowska E., Gryglewicz G., Diez M.A. (2013). Kinetics and equilibrium study of phenol adsorption on nitrogen-enriched activated carbons. Fuel.

[11] Li L., Quinlivan P.A., Knappe D.R.U. (2002). Effects of activated carbon surface chemistry and pore structure on the adsorption of organic contaminants from aqueous solution. Carbon.

[12] Lu A.H., Zheng J.T. (2001). Study of Microstructure of High-Surface-Area Polyacrylonitrile Activated Carbon Fibers. J. Colloid Interface Sci..

[13] Gibaud A., Xue J.S., Dahn J.R. (1996). A small angle X-ray scattering study of carbons made from pyrolyzed sugar. Carbon.

[14] Liu Y., Di D., Bai Q., Li J., Chen Z., Lou S., Ye H. (2011). Preparative separation and purification of rebaudioside a from steviol glycosides using mixed-mode macroporous adsorption resins. J. Agric. Food Chem..

[15] Anandkumar J., Mandal B. (2009). Removal of Cr(VI) from aqueous solution using Bael fruit (*Aegle marmelos correa*) shell as an adsorbent. J. Hazard. Mater..

[16] He J., Guang W., Li G.H., Mi N., Xue H.X., Tian H.J., Lu C.W., Gao X.D. (2005). Adsorptive behaviors of phenolic compounds in sediments of the yellow river. J. Agro-Environ. Sci..

[17] Liu Y.F., Bai Q.Q., Lou S., Di D.L., Li J.T., Guo M. (2012). Adsorption characteristics of (–)-epigallocatechin gallate and caffeine in the extract of waste tea on macroporous adsorption resins functionalized with chloromethyl, amino, and phenylamino groups. Agric. Food Chem..

[18] Mahmoud H.R., Ibrahim S.M., El-Molla S.A. (2016). Textile dye removal from aqueous solutions using cheap MgO nanomaterials: Adsorption kinetics, isotherm studies and thermodynamics. Adv. Powder Technol..

[19] Wang D.W., Liu H.C., Song C.C., Wei C.G., Liu T.T. (2013). Ultrasonic-assisted extraction and characterization of potato starch. Food Sci..

